# Epidemiology of injuries among Malaysian adolescent karate athletes: A cross-sectional study

**DOI:** 10.51866/oa.632

**Published:** 2024-10-19

**Authors:** Mohamad Azwan Aziz, Punitha Kunabal

**Affiliations:** 1 MBBS, MRCPi, MFSEM, FIPM, Sports Medicine Department, University Malaya Medical Centre, Jln Profesor Diraja Ungku Aziz, Kuala Lumpur, Malaysia.; 2 Sports Medicine Unit, Faculty of Medicine, University of Malaya, Kuala Lumpur, Malaysia. Email: letsgetfitdrazwan@gmail.com; 3 MBBS, Sports Medicine Department, University Malaya Medical Centre, Jln Profesor Diraja Ungku Aziz, Kuala Lumpur, Malaysia.; 4 Sports Medicine Unit, Faculty of Medicine, University of Malaya, Kuala Lumpur, Malaysia.

**Keywords:** Karate, Injury prevention, Athletic Injuries, Adolescent

## Abstract

**Introduction:**

Despite the wide range of injuries sustained by adolescents during karate, limited studies have investigated the type and frequency of sports injuries among adolescent karate athletes. This study would be the first to examine the epidemiology of injuries among adolescent karate athletes. Its objective was to describe the incidence of injuries throughout the 2023 season and the pattern of injuries among Malaysian adolescent karate athletes.

**Methods:**

From 1 January to 31 December 2023, this cross-sectional study examined 119 Malaysian adolescent karate athletes aged 13-19 years. Data on demographic characteristics and patterns of injuries were collected using a standardised form and analysed using SPSS version 26.

**Results:**

The incidence of injuries was 22.3 injuries per 1000 athlete exposures. Specifically, the incidence of injuries during training was 15.34 injuries per 1000 athlete exposures, while that during competition was 106.6 injuries per 1000 athlete exposures. The majority of the injuries were mild (n=113, 87.6%), while the minority were severe (n=6, 4.7%). The injuries most commonly occurred in the head and neck (n=31, 24%), followed by the ankle (n=13, 10.1%) and foot (n=14, 10.9%).

**Conclusion:**

There is a need to explore the extrinsic and intrinsic risk factors of injuries among adolescent karate athletes in Malaysia, as the incidence of injuries is high during competition. Additionally, it is important to educate athletes and supporting sports science members about injury prevention programmes.

## Introduction

Karate is the most widely used form of martial art worldwide. It is a Japanese martial art that has been included in Olympic sports. Its main technique is standing-based striking, including punches and kicks. With 190 member nations, the World Karate Federation (WKF) is one of the largest organisations in karate.^1^ Karate has two main categories: (i) sparring (kumite) and (ii) forms (kata). Individual kumite is subdivided into age and weight divisions. For juniors, cadets and children, the strict regulations of the WKF kumite rules allow sweeps, throws and strikes to the trunk but prohibit contact strikes to the head and face.^2^ These strict rules aim to reduce severe head and neck injuries in the paediatric population. WKF-approved protective equipment is mandatory in kumite to reduce the risk of injuries. This includes body protectors (plus chest protectors for female athletes), mouthguards, hand protectors (mitts) and shin and foot protectors.

A study in Malaysia found that the most common intention for joining martial arts was to improve physical fitness and engage in social activities such as meeting new friends and networking.^3^ While martial arts emphasise physical fitness, sports injuries have emerged as a significant public health concern in developed nations, accounting for 19%-59% of all paediatric injuries.^4-6^ To our knowledge, limited studies have evaluated the epidemiology of injuries among adolescent karate athletes. According to the World Health Organization, adolescents are defined as individuals aged 10-19 years.^7^ Adolescence is a crucial stage of life, and injuries during this period can hinder the growth and development of athletes in the future. In 2000, Zetaruk et al. examined 68 karate athletes in Plymouth and found that 28% of these athletes sustained at least one injury during the 1995-1996 season.^8^ Another prospective cohort study reported an overall incidence of 148.0 injuries per 1000 athlete exposures (AEs).^9^ Arriaza et al. showed a low incidence of 30.0 injuries per 1000 AEs in 848 elite karate athletes.^10^ No previous studies have examined the Malaysian population. This study would be the first to examine the epidemiology of injuries among Malaysian adolescent karate athletes. Its primary objective was to describe the incidence of injuries throughout the 2023 season and the pattern of injuries among Malaysian adolescent karate athletes. The secondary objective was to examine the relationship between intrinsic risk factors [age; sex; years of training; anthropometric measurements such as weight, height, body mass index (BMI), skeletal muscle mass and body fat percentage; and physical fitness parameters such as muscle strength, core endurance and cardiorespiratory fitness] and injuries.

## Methods

### Study design

This study adopted a cross-sectional design and was conducted at eight karate training facilities in the Klang Valley selected via convenience sampling. The calculated sample size was 153, determined using the Epitools calculator based on an estimated population proportion of 10% and a dropout rate of 10%.^11^ However, only 119 athletes participated. The target sample size could not be achieved due to difficulties in recruitment. All athletes aged 13—19 years were recruited regardless of their sex, skill level or belt rank. At the training centres, each athlete received personal information about the study and the consent form. Prior to the study’s execution, informed consent was obtained from participants; in cases of individuals under the age of 18 years, consent was sought from their parents or legal guardians. Athletes who did not consent and athletes whose parents and guardians did not consent were excluded from this study. These procedures were completed at the training facilities by the principal investigator. Ethical approval for the conduct of the study was obtained from the University of Malaya Research Ethics Committee (UM. TNC2/UMREC_2256).

### Data collection

Data were gathered from 1 January to 31 December 2023. With the help of the lead investigator, participants completed a Google Form containing questions about their demographics and injury history. The questionnaire was adapted from the Australian Sports Injury Data Dictionary.^12^ Age, date of birth, sex, race, belt rank, years of experience, medical history, history of injury, type of injury, mechanism of injury, site of injury, time of occurrence (during warm-up, training or competition), number of training hours per week, use of protective gear and time lost as a result of injury were among the data collected. The researcher asked participants to provide a retrospective description of any injuries they had sustained in the preceding year during the data collection period. Each finding was documented using a standardised clinical research form.

### Statistical analysis

Age and baseline anthropometric measurements (weight, height, BMI, skeletal muscle mass and body fat percentage) were presented as means and standard deviations. Demographic data (sex, dominant hand, belt rank and years of training), and injury data (site of injury, type of injury, severity of injury and mechanism of injury) as frequencies and percentages. The site and type of injury during competition, training and warm-up were compared. The incidence of injuries was measured per 1000 AEs.^11^ AE was calculated based on the total number of training days and the total number of competition days. The following formula was used: incidence of injuries = number of injuries/number of athletes × 1000 AEs.

A binary logistic regression analysis was performed to explore the intrinsic risk factors of injuries (age, sex, years of training, anthropometric measurements and physical fitness parameters). P-values less than 0.05 were considered significant, and odds ratios were calculated. All analyses were conducted using SPSS version 26 (IBM Corp. Released 2019. IBM SPSS Statistics for Windows, Version 26.0. Armonk, NY: IBM Corp)

## Results

A total of 119 adolescent karate athletes participated in this study. Their ages ranged from 13 to 19 years, with a mean age of 15.6±1.8 years. Among the participants, 67.2% were boys; 52.1% had karate experience of 6—10 years; and 51.3% were brown belters. The baseline characteristics of the participants are outlined in Table 1.

**Table 1:** Baseline characteristics, anthropometric measurements and physical fitness parameters among the 119 adolescent karate athletes.

**Characteristics**	
**Sex**	
Male	39 (32.8)^†^
Female	80 (67.2)^†^
**Karate belt**	
Black	35 (29.4)^†^
Blue	8 (6.7)^†^
Brown	61 (51.3)^†^
Green	2 (1.7)^†^
Purple	9 (7.6)^†^
Yellow	4 (3.24)^†^
**Experience in training (year)**	
1-5	33 (27.7)^†^
6-10	62 (52.1)^†^
11-15	19 (16)^†^
16-20	5 (4.2)^†^
**Experience in training (year)**	15.6 (1.8)^*^
**Anthropometric measurements**	15.6 (1.8)^*^
Weight (kg)	165.7 (9.1)^*^
Height (cm)	59.4 (14.3)^*^
Body mass index (kg/m^2^)	21.5 (4.5)^*^
Skeletal muscle mass (kg)	24.1 (5.5)^*^
Body fat percentage (%)	24.8 (11.2)^*^
**Physical fitness parameters**	15.6 (1.8)^*^
Dominant hand grip strength (kg)	30.7 (9.3)^*^
1-min sit-up test count (repetition)	31.4 (8.5)^*^
VO_2_ peak (mL/kg/min)	59.0 (12.4)^*^

* Expressed as mean (standard deviation)

† Expressed as frequency (percentage)

Among the 119 participants, 58.8% (n=70) sustained at least one injury. The total number of injuries reported was 129. The incidence of injuries was 22.3 injuries per 1000 AEs. Specifically, the incidence of injuries during training was 15.34 injuries per 1000 AEs, while that during competition was 106.6 injuries per 1000 AEs. The mean age of the participants who sustained injuries was 15.9±1.79 years. The majority of the injuries were reported at the age of 14-15 years (n=27, 38.6%) and 16-17 years (n=22, 31.4%). The male participants reported a higher incidence of injuries (n=48, 68.6%). All participants who sustained injuries had a history of injury (100%). Throughout the season, 45.7% sustained only one injury, while 31.4% sustained two injuries. The epidemiology of the injuries is outlined in Table 2.

**Table 2:** Epidemiology of the injuries sustained by 70 athletes.

**Epidemiology**	
Age (year)	15.9 (1.79)^*^
13	5 (7.1)1^*^
14-15	27 (38.6)^†^
16-17	22 (31.4)^†^
18-19	16 (22.9)^†^
**Sex**	
Male	48 (68.6)^†^
Female	22 (31.4)^†^
**Karate belt**	
Black	25 (35.7)^†^
Blue	2 (2.9)^†^
Brown	38 (54.3)^†^
Purple	4 (5.7)1^*^
Yellow	1 (1.4)^*^
Green	0(0)^*^
**Experience in training (year)**	
1-5	12 (17.1)^†^
6-10	40 (57.1)^†^
11-15	15 (21.4)^†^
16-20	3 (4.3)^†^
**History of injury**	70 (100)^†^
**Number of injury**	
1	32 (45.7)^†^
2	22 (31.4)^†^
3	13 (18.6)^†^
4	1 (1.4)^†^
5	2 (2.9)^†^

* Expressed as mean (standard deviation)

† Expressed as frequency (percentage)

Most injuries occurred during training (n=83, 64.3%). The most commonly reported mechanism of injury was striking by the opponent (n=62, 48.1%). The majority of the injuries were mild (n=113, 87.6%), while the minority were severe (n=6, 4.7%). The injuries most frequently occurred in the head and neck (n=31, 24%), followed by the ankle (n=13, 10.1%) and foot (n=14, 10.9%). The most common type of injury was sprain/strain (n=55, 42.6%). The pattern of the injuries is outlined in Tables 3-5.

**Table 3:** Patterns of 129 injuries: Mechanism of injuries, time lost due to injuries and time of occurrence.

Patterns	n	%
**Time of occurrence**		
During competition	40	31
During training	83	64.3
During warm-up	6	4.7
**Total days**		
Training	5408	93.5
Competition	375	6.5
**Usage of personal protective equipment**		
Yes	92	71.3
No	37	28.7
**Mechanism of injury**		
Contact with a player	11	8.5
Contact with an object	5	3.9
Awkward landing	1	0.8
Fall	6	4.7
Jumping	5	3.9
Others	13	10.1
Overuse	11	8.5
Slip/trip	5	3.9
Striking by another player	62	48.1
Twisting	10	7.8
**Time lost due to injury**		
Mild (0-7 days)	113	87.6
Moderate (8-28 days)	10	7.8
Severe (>28 days)	6	4.7

**Table 4:** Comparison of the site of injuries during competition, training and warm-up.

Site	Total n (%)	Competition n (%)	Training n (%)	Warm-up n (%)
Abdomen	4 (3.1)	1 (2.5)	2 (2.4)	1 (16.7)
Ankle	13 (10.1)	2 (5)	11 (13.3)	0 (0)
Chest	7 (5.4)	4 (10)	3 (3.6)	0 (0)
Foot	14 (10.9)	1 (2.5)	13 (15.7)	0 (0)
Forearm	4 (3.1)	0 (0)	4 (4.8)	0 (0)
Head and neck	31 (24)	18 (45)	13 (15.7)	0 (0)
Hip and groin	9 (7)	3 (7.5)	5 (6)	1 (16.7)
Knee	9 (7)	2 (5)	6 (7.2)	1 (16.7)
Lower leg	7 (5.4)	1 (2.5)	6 (7.2)	0 (0)
Shoulder	1 (0.8)	0 (0)	1 (1.2)	0 (0)
Spine	4 (3.1)	0 (0)	4 (4.8)	0 (0)
Thigh	10 (7.8)	2 (5)	5 (6)	3 (50)
Upper arm	1 (0.8)	1 (2.5)	0 (0)	0 (0)
Wrist	15 (11.6)	5 (12.5)	10 (12)	0 (0)

**Table 5:** Comparison of the type of injuries during competition, training and warm-up.

Type	Total n (%)	Competition n (%)	Training n (%)	Warm-up n (%)
Abrasion wound	12 (9.3)	2 (5)	10 (12)	0 (0)
Bruise/contusion	25 (19.4)	12 (30)	13 (15.7)	0 (0)
Concussion	2 (1.6)	1 (2.5)	1 (1.2)	0 (0)
Inflammation/swelling	9 (7)	3 (7.5)	6 (7.2)	0 (0)
Loss of consciousness	6 (4.7)	5 (12.5)	1 (1.2)	0 (0)
Open wound/laceration/cut	3 (2.3)	1 (2.5)	2 (2.4)	0 (0)
Others	9 (7)	4 (10)	5 (6)	0 (0)
Overuse injury	6 (4.7)	1 (2.5)	4 (4.8)	1 (16.7)
Chest wall injury	2 (1.6)	2 (5)	0 (0)	0 (0)
Sprain/strain	55 (42.6)	9 (22.5)	41 (49.4)	5 (83.3)

The binary logistic regression analysis showed no significant correlation between the intrinsic risk factors (age, sex, years of training, anthropometric measurements and physical fitness parameters) and injuries (Table 6).

**Table 6:** 

Intrinsic risk factors	Odds ratio	P-value	Confidence interval
Age (year)	0.983	0.910	0.733-1.318
**Sex**			
Male	2.64	0.673	0.029-241.7
**Experience in training (year)**			
1-5	0.337	0.231	0.132-0.861
6-10	1.392	0.625	0.369-5.25
11-15	0.696	0.735	0.086-5.66
**Anthropometric measurements**			
Height (cm)	1.09	0.892	0.774-1.342
Weight (kg)	0.895	0.633	0.569-1.410
Skeletal muscle mass (kg)	1.38	0.234	0.810-2.363
Body fat percentage (%)	1.16	0.136	0.953-1.419
Body mass index (kg/m^2^)	0.961	0.940	0.338-2.733
**Physical fitness parameters**			
Hand grip strength (kg)	1.028	0.565	0.936-1.129
1-min sit-up rest count (per minute)	1.010	0.714	0.958-1.064
Step-up test heart rate (beat per min)	1.003	0.951	0.923-1.089
VO_2_ max (mL/kg/min)	1.047	0.703	0.828 1.324

Odds ratios with 95% confidence intervals were calculated for various intrinsic risk factors. The reference categories for the categorical variables were as follows:

- Sex: The reference category was female.
- Experience in training: The reference category was >16 years of experience.

For age, height, weight, skeletal muscle mass, body fat percentage, body mass index, hand grip strength, 1-min sit-up test count, step-up test heart rate and VO2 max, odds ratios represent the changes in the odds associated with a 1-unit increase in each variable.

## Discussion

Many studies conducted on the epidemiology of injuries among karate athletes have focused on injuries sustained during competition and on senior athletes.^13^ To our knowledge, the present study is the first to examine the epidemiology of injuries among Malaysian adolescent karate athletes. The overall incidence of injuries in our study was low at 22.3 injuries per 1000 AEs. However, when compared to other international reports, the incidence of injuries during competition is high at 106.6 injuries per 1000 AEs in this study. An epidemiological study conducted among adolescent karate athletes during the World Karate Championships from 2009 to 2015 reported an incidence of 41.4 injuries per 1000 AEs.^14^ Another in-competition injury surveillance study performed among 1733 adolescent athletes showed an incidence of 45.26 injuries per 1000 AEs.^11^ Arriaza et al. reported an incidence ranging from 41.4 to 59.8 injuries per 1000 AEs during three World Karate Championships.^10^ One cross-sectional study found that the incidence of karate injuries during training was 202 injuries per 1000 AEs.^15^ All of these studies have a comparable age demographic to our study. Destombe et al. reported that 28.8% of injuries occurred during training and 75.9% during competition.^16^ Although our study noted a high incidence of injuries during competition, most of them were mild. In this study, the high incidence of injuries can be attributed to noncompliance with personal protective equipment. Other studies have focused on elite athletes, who generally have better injury prevention knowledge than our amateur athletes. In our study, the incidence of injuries was higher among the participants aged 14-15 years. Cierna and Lystad reported a higher incidence among participants aged 12-17 years (Incidence Rate Ratio=2.85, Confident Interval=2.07-3.93) than among those aged 6-12 years.^11^ The more experienced participants in our study had a low incidence of injuries, in contrast to some reports showing an increased incidence of injuries in athletes with longer experience.^8,16,17^ This may be because more experienced karate athletes are more adept at blocking opponents’ strikes and positioning themselves effectively during attacks, which help reduce their risk of injuries.^18^

Our study found a high incidence of injuries in the lower limbs (48.1%), echoing other reports.^16,19^ Lower limb injuries were commonly reported during training (55.1%), while head and neck injuries were frequently reported during competition. In other studies on injury surveillance during international competition, the head and neck regions were the commonly reported sites of injuries.^11,17^ In a previous meta-analysis, the head and neck were also the most commonly injured body regions (median=57.9%).^13^ High scores are awarded for targeting the head of opponents, which places the head and neck at a higher risk of injuries during competition.^20^ Destombe et al. reported a high incidence of injuries in the lower limbs (35%) among 186 individuals from three karate clubs.^16^ Similar to our findings, the incidence of lower limb injuries was high during training (38% vs 25%). Karate athletes are at risk of injuring their lower limbs, especially the weight-bearing limb, due to the repetitive nature of kicks, turns, throws, shuffles and lunges. During training sessions, repetitive movements and fatigue due to long hours of training contribute to an increased risk of acute and overuse injuries. Understanding the prevalence of injuries during training and competition highlights the need for targeted preventive measures. Specifically, preventing head and neck injuries should be prioritised during competition, while preventing lower limb injuries should be emphasised during training sessions. Injury prevention strategies during training should include balance and strength training for the lower limbs, while those during competition should enforce the usage of personal protective equipment.

In our study, strains and sprains were the most common types of injuries seen (40.9%), with a higher incidence during training (47.2%) than during competition (21.4%). Similarly, Destombe et al. reported a high incidence of strains during training (21% vs 15%).^16^ They found that strikes were responsible for 87.5% of sprains and 84.6% of strains. In contrast, Arriaza and Leyes reported a low incidence of sprains (3.5%) and a high incidence of contusions (50.3%), epistaxis (16.2%) and lacerations (13.7%).^21^ In our study, contusions or bruises were common during competition. Halabchi et al. and Rahimi et al. noted similar findings, reporting an incidence of contusions of 43.6% and 60%, respectively.^16,22^

To date, limited studies have evaluated the relationship of anthropometric measurements and physical fitness parameters with injuries among karate athletes. In the current study, the binary regression analysis failed to demonstrate a significant association between the intrinsic risk factors (anthropometric measurements and physical fitness parameters) and injuries. This could be due to our small sample size. A previous study reported that age, training volume and BMI were important predictors of acute and overuse injuries in martial arts.^23^ Younger athletes sustained fewer injuries, as they tended to have lower weight. Vitale et al. also found that higher weight increased the risk of injuries.^23^ A military study demonstrated that athletes with poor cardiorespiratory endurance and muscle endurance were more prone to injuries and therefore concluded that improving physical fitness can reduce the risk of injuries.^24^ Low fitness levels can lead to easy fatigability, increasing the risk of injuries. Further longitudinal studies are needed to explore this association among Malaysian adolescent karate athletes.

## Strengths and limitations

Despite the wide range of injuries among adolescent karate athletes, limited studies have investigated the type and frequency of sports injuries. This information may help prevent, diagnose and treat sports injuries. The present study is the first in Malaysia to evaluate the incidence of injuries among adolescent karate athletes. The findings offer insights into injury prevention in karate training programmes. For instance, the higher incidence of lower limb injuries could be addressed by incorporating proprioceptive training, cardiovascular fitness and muscle endurance into training programmes. During competitions, it is crucial to raise athletes’ awareness about the importance of using personal protective equipment, such as mouthguards, chest protectors, groin guards and hand mitts, to prevent or reduce the risk of injuries. This study also provides strong evidence for policymakers to strengthen regulations regarding the use of face shields for adolescents, thereby helping to reduce the risk of head and neck injuries. However, we acknowledge a few limitations in this study. The focus on karate athletes from the Klang Valley may limit the generalisability of the results to broader populations. Furthermore, recall bias is possible, as injury incidence and training history were based solely on self-reported data.

## Conclusion

The overall incidence of injuries among adolescent karate athletes in Malaysia is low compared to international reports. However, the incidence of injuries during competition is high. There is a need to explore the extrinsic and intrinsic risk factors of injuries and educate athletes and supporting sports science members about injury prevention programmes. Approximately 86.1% of athletes sustain mild injuries. The recurrence rate of injuries is high (53.3%). The incidence of injuries is higher in the lower limbs during training and in the head and neck during competition. These findings can inform the development of holistic strategies for injury prevention during training and competition.

## Data sharing statement

Data are available only upon request.

**How does this paper make a difference in general practice?**

- In Malaysia, this study is one of the earliest to explore the incidence of injuries among adolescent karate athletes during training and competition.
- This information can aid in better planning for injury prevention programmes for this age group.
